# A Comprehensive Review on MAPK: A Promising Therapeutic Target in Cancer

**DOI:** 10.3390/cancers11101618

**Published:** 2019-10-22

**Authors:** Cornelia Braicu, Mihail Buse, Constantin Busuioc, Rares Drula, Diana Gulei, Lajos Raduly, Alexandru Rusu, Alexandru Irimie, Atanas G. Atanasov, Ondrej Slaby, Calin Ionescu, Ioana Berindan-Neagoe

**Affiliations:** 1 Research Center for Functional Genomics, Biomedicine and Translational Medicine, Iuliu Hatieganu University of Medicine and Pharmacy, 23 Marinescu Street, 40015 Cluj-Napoca, Romania; braicucornelia@yahoo.com (C.B.); busuioc.constantin@gmail.com (C.B.); raduly.lajos78@gmail.com (L.R.); ioananeagoe29@gmail.com (I.B.-N.); 2 MEDFUTURE-Research Center for Advanced Medicine, Iuliu Hatieganu University of Medicine and Pharmacy, 23 Marinescu Street, 40015 Cluj-Napoca, Romania; buse.mihail@umfcluj.ro (M.B.); diana.gulei@umfcluj.ro (D.G.); 3 Biozoon GmbH, D-27572 Bremerhaven, Germany; rusu@biozoon.de; 4 Department of Surgery, The Oncology Institute “Prof. Dr. Ion Chiricuta”, 40015 Cluj-Napoca, Romania; airimie@umfcluj.ro; 5 Department of Surgical Oncology and Gynecological Oncology, Iuliu Hatieganu University of Medicine and Pharmacy, 40015 Cluj-Napoca, Romania; 6 Department of Pharmacognosy, University of Vienna, Althanstrasse 14, 1090 Vienna, Austria; atanas.atanasov@univie.ac.at; 7 Institute of Genetics and Animal Breeding of the Polish Academy of Sciences, Jastrzebiec, 05-552 Magdalenka, Poland; 8 Institute of Neurobiology, Bulgarian Academy of Sciences, 23 Acad. G. Bonchev Str., 1113 Sofia, Bulgaria; 9 Central European Institute of Technology, Masaryk University, 601 77 Brno, Czech Republic; on.slaby@gmail.com; 10 Department of Comprehensive Cancer Care, Masaryk Memorial Cancer Institute, 601 77 Brno, Czech Republic; 11 5th Surgical Department, Municipal Hospital, 400139, Cluj-Napoca, Romania; 12 Department of Surgery, Iuliu Hatieganu University of Medicine and Pharmacy, 23 Marinescu Street, 40015 Cluj-Napoca, Romania; 13 Department of Functional Genomics and Experimental Pathology, The Oncology Institute Prof. Dr. Ion Chiricuta, Republicii 34 Street, 400015 Cluj-Napoca, Romania

**Keywords:** cancer, MAPK, molecular mechanisms, drug resistance

## Abstract

The mitogen-activated protein kinase (MAPK) pathway is an important bridge in the switch from extracellular signals to intracellular responses. Alterations of signaling cascades are found in various diseases, including cancer, as a result of genetic and epigenetic changes. Numerous studies focused on both the homeostatic and the pathologic conduct of MAPK signaling; however, there is still much to be deciphered in terms of regulation and action models in both preclinical and clinical research. MAPK has implications in the response to cancer therapy, particularly the activation of the compensatory pathways in response to experimental MAPK inhibition. The present paper discusses new insights into MAPK as a complex cell signaling pathway with roles in the sustenance of cellular normal conduit, response to cancer therapy, and activation of compensatory pathways. Unfortunately, most MAPK inhibitors trigger resistance due to the activation of compensatory feed-back loops in tumor cells and tumor microenvironment components. Therefore, novel combinatorial therapies have to be implemented for cancer management in order to restrict the possibility of alternative pathway activation, as a perspective for developing novel therapies based on integration in translational studies.

## 1. Introduction

Cancer stands as one of the greatest challenges to global health. Estimates suggest more than 1,735,350 new cases for 2018 and 609,640 deaths annually [1]. The diagnostic rate increased in recent years, as did the overall life expectancy of patients due to recent advances in novel therapies. Molecular diagnostics made it possible to subcategorize each cancer type based on the mutational status of several important genes [2,3], indicating the molecular patterns that are malfunctioning, thereby allowing efficient intervention with targeted therapeutics by inhibiting specific biological pathways of tumor cells [2]. Cancer is commonly correlated with the acquisition of several mutations that disrupt key signaling pathways [2,4,5,6,7]. Cellular signaling pathways are structured as continuously communicating modular networks. Pathway components interact in a switch-like manner, with the interaction between two proteins resulting in either the direct or the indirect activation or inhibition of the subsequent factor [8,9,10]. The pathologic conduit of different signaling pathways is sustained by genetic, transcriptomic, and epigenetic alterations with implications in different mechanisms, such as cellular fate [6,7,11,12].

As molecular diagnostics tools became widely clinically accessible, the identification of specific mutational patterns of cancers became a useful method of stratifying patient cohorts with similar alterations, used for assessing the most efficient treatment [4,7,10,13,14,15]. In spite of this progress, the resistance to therapy still represents a major problem, a commonly occurring effect in patients manifested after first-line treatment. Targeted therapy, using different small molecules that act as inhibitors of key signaling steps, can induce resistance, in some cases even from the first doses. Resistance occurs as an evolutionary effect of positively selecting tumor cells with mechanisms that can compensate the specific targeted pathway [3,9,16].

The mitogen-activated protein kinase (MAPK) is one such complex interconnected signaling cascade with frequent involvement in oncogenesis, tumor progression, and drug resistance. The MAPK family consists of a large number of kinases altered in cancers and against which many targeted therapies were developed. Resistance to MAPK inhibitors is a current problem, particular due to the high degree of interactions and possible compensatory responses. Thus, in this review, we discuss the many implications of the MAPK pathways in cancer, with a particular focus on the regulation of tumor signaling through emphasis of MAPK crosstalk with key signaling pathways in pathological conditions.

## 2. Physiological Roles of the MAPK Signaling Pathway

**Figure 1:**
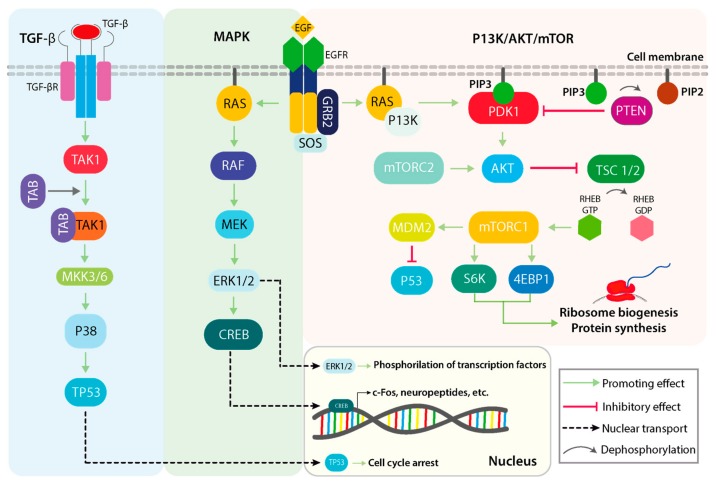
Parallel outline of several physiological roles of the TGFβ/p38, mitogen-activated protein kinase (MAPK), and P13k/AKT/mTOR signaling pathways. The p38 mitogen-activated kinase can be activated following upstream cytokine stimulation of the TGFβ pathway, which can subsequently activate TP53 in normal physiological conditions. TGFβ activation of p38 is not dependent on canonical SMAD signaling, but rather on the *TAB/TAK1* complex and the *MKK3/6* mitogen-activated protein kinase kinases. The canonical MAPK kinase pathway initiates with an extracellular stimulus in the form of growth factors (GFs) that bind and activate receptor tyrosine kinases (RTKs) on the cell membrane. Downstream activation of *RAS, RAF* and *MEK* in that order converge in the activation of the *ERK1/2* transcription factor activator. The P13K/AKT/mTOR cascade can also be activated via RTKs and RAS, and its main implications are related to metabolic signaling and protein synthesis that sustain cell growth. TGFβ: transforming growth factor beta 1; p38: p38 kinase; P13k: phosphoinositide-3-kinase; AKT: v-akt murine thymoma viral oncogene homolog 1; mTOR: mechanistic target of rapamycin kinase; TAB: TGF-beta activated kinase 1 binding protein 2; TAK1: TGF-beta activated kinase 1; MKK3/6: mitogen-activated protein kinase kinase 3; RAS: small G-protein; RAF: Raf oncogene; MEK: MAP kinse-ERK kinase; RTKs: Receptor tyrosine kinases.

The framework of a signaling cascade such as MAPK is complex, with many interacting pathways and constant crosstalk, which are each subjected to fine-tuning and switch-like activations of regulatory factors (Figure 1). As an overview, the MAPK pathways converge in the amplification of key molecules that sustain cell proliferation, growth, and survival processes [17,18]. The outline of the MAPK signaling cascade consists of the interaction of one or more growth factors (GFs) with their specific growth factor receptors (GFRs). Generally, GFs bind and activate transmembrane glycoproteins of the receptor tyrosine kinase (RTK) family and activate the signal transduction cascade, followed by signal transduction through cytosolic intermediates, and finally the transcription/translation regulation of effector genes [19]. These are mainly represented by growth factor receptors, which promote, following their activation, the downstream signal transduction [19]. One such example consists of the epithelial growth factor (EGF), which targets its high-affinity membrane receptor (EGFR: epidermal growth factor receptor) [20].

The first line of cytosolic intermediates that activate the phosphorylation cascade of the MAPK pathway are represented by the RAS superfamily of GTPases, which comprise over 150 small G-proteins, such as HRAS, KRAS, NRAS, and others [21]. Subsequent to EGFR activation, the RAS GTPase is activated with the help of EGFR-associated of nucleotide exchange factor Son of Sevenless 1 (SOS1) [22]. SOS determines the rapid conversion of GTP to GDP, which is a limiting condition for the formation of RAS-GTP, the active form of RAS [22]. RAF is the downstream effector of RAS and, thus, it is dependent on the interaction with an activated RAS. The RAF family includes several variants (e.g., ARAF, BRAF, CRAF) [23,24], all of which consist of serine/threonine kinases responsible for the pathway progression by activating MEK (MAP kinse-ERK kinase) and ERK1/2 (Extracellular signal-regulated kinases). The activation cascade is in the following order: MAPKKK (Mitogen-activated protein kinase kinase kinases, represented by RAF and its variants), followed by MAPK kinase (MAPKK: MEK1/2/3/4/5/6/7), and finally the MAPK. There are three main classical MAPKs with different isoforms ERKs (with ERK1 and ERK2 isoforms), JNKs (c-Jun N-terminal kinases, with JNK1, JNK2, and JNK3 isoforms), and p38 MAPKs (with p38α, p38β, p38γ, and p38δ isoforms) [24,25,26]. Both MEK and ERK1/2 are involved in a wide range of processes, such as cell survival, proliferation, and differentiation, all of which are dependent on the phosphorylated targets of MEK and ERK1/2 respectively. ERK1/2 especially displays variability in its phosphorylation targets, independent of cellular location and compartment. In the nuclease, ERK1/2 can activate transcription factors, such as CREB (cAMP response element-binding protein), as well as c-Myc (transcriptional regulator Myc-like) and NF-κB (nuclear factor kappa B). This makes ERK1/2 an important anti-tumor target [24,25,26].

Complementary signaling pathways display similar architecture to the MAPK pathways, in the form that all initiate post interaction with an extracellular stimulus (either GFs or cytokines) and converge in nuclear translocation of specific factors that promote gene expression. With regard to the regulatory activity of the MAPK pathway, other relevant pathways are *P13k/AKT/mTOR* (phosphoinositide-3-kinase/v-akt murine thymoma viral oncogene homolog 1/mechanistic target of rapamycin kinase) and TGFβ (transforming growth factor beta) signaling, both of which display varying degrees of interaction and cumulative signal transduction [8]. The nuclear translocation of MAPK signaling constituents may be considered as a significant regulatory system of key cellular processes, representing a possible therapeutic intervention [8].

## 3. MAPK-Signaling Crosstalk and Pathologic Deregulations in Cancer

Cancer-associated alterations of MAPK signaling arise as a result of its effectors suffering mutations that affect the functionality and, therein, the progression of the signaling cascade in both forms of constitutive activation and continuous signal transduction. As previously mentioned, the MAPK pathways are an extensive regulatory network consisting of a series of crosstalking and compensatory pathways implicated in transducing distress, thereby affecting growth signals and cellular metabolism [8]. Evidence indicates that these effectors are involved in key roles of cancer progression and therapeutic resistance [8]. Some relevant examples are summarized in Table 1. The majority of solid tumors are explicitly characterized by their mutations in the *RAS*/*RAF*/*MEK*/*ERK* genes of the signaling pathway [27]. Mutations in the *BRAF* (B-Raf proto-oncogene serine/threonine kinase) and *RAS* family genes (*KRAS* and *NRAS*) are frequent (e.g., *RAS-*small G-protein: 15–29% in melanoma, colorectal: 34.1%, lung cancer: 12–30% and *BRAF*: 50–60 in melanoma; colorectal: 5–20%, lung cancer: 4%), whilst mutations in *MEK (*MAP kinse-ERK kinase) or *ERK* (extracellular regulated MAP kinase) are identified less (melanoma 3–8%, colorectal 3%) or rarely, respectively [28]. Moreover, mutations also occur in the genes coding for the tyrosine kinase receptors (*EGFR, c-MET, c-KIT*), in addition to the main signal transduction cascade members previously mentioned. Other proteins, such as the Sprouty/Spred family [29] and downstream kinases (*BRAF*) can be targeted and, subsequently, altered. These exhibited modifications represent a promising starting point for experimental therapeutic strategies [28]. The genes presented as incidences of mutations accentuate that downstream inhibition of these targets has desirable prospects; for example, ERK1/2 kinase inhibitor ulixertinib passed phase I clinical trials with favorable pharmacokinetics and a dose-escalation model for solid tumor malignancies [30].

According to the data retrieved from cancer mutation databases, around 30% of human solid tumors are characterized by a mutation in a *RAS* gene [31]. These mutations are indicators of cancer aggressiveness and are commonly correlated with unfavorable prognosis in patients [32]. RAS acts as an activator for both MAPK and P13k/AKT/mTOR pathways, emphasizing its important role as a regulator for all the involved pathways and the consequences of its alterations in the case of cancer. The dynamic interaction between RAS/ERK and RAS/PI3K is characterized by both positive and negative feedback loops, assuring a bidirectional communication with other pathways [28]. This crosstalk continues to function in different cancers, although in the altered form, such as in the case of in ulcerative colitis-associated colon cancer, where an increase in the activity in ERK and PI3K/AKT pathways was observed [33]. This makes the RAS kinase subfamily members one of the first therapeutic targets investigated as possible targets in the MAPK pathway. HRAS mutations are related to the hyperactivation of the RAS and the mTOR pathways. Therefore, HRAS mutations sensitize the response to MEK inhibitors (AZD6244, MEK162 and PD0325901), leading to a significant reduction of cell proliferation [34]. On the other hand, RAS can lead to the suppression of PTEN (phosphatase and tensin homolog), the main inhibitor of P13K activation. PTEN subsequently dephosphorylates PIP3 to PIP2, an event which is important in regulating the pathway; *PTEN* mutations also affect the response to ERK inhibitors. Moreover, PTEN also inhibits AKT and, subsequently, mTORC1 activation [35,36].

The PTEN mutational status affects the response to combined therapy based on MEK and mTOR inhibitors in cancer [37], a fact that needs to be further investigated in the context of personalized treatment. PTEN proved to be a vital factor for promoting the response to MAPK inhibitors in myeloid leukemia, as PTEN regulates *EGR1* expression and contributes to the cytokine sensitivity when treated with MAPK inhibitors [38]. PTEN loss and activation of KRAS (Kirsten rat sarcoma viral oncogene homolog) are correlated with cytoskeleton alteration, and they act as possible therapeutic targets which would allow testing of new compounds for more specific targeted therapies, having the capacity to modulate the PI3K and RAS/MAPK pathways [39]. To further consolidate this idea, experimental data on normal breast cells showed that PTEN inhibition is related to the activation of KRAS, with an impact on PI3K/AKT/mTOR and RAS/MAPK signaling, confirming the interconnection between the cellular pathways [39]. The loss of PTEN, leading to RAS/MAPK activation, was proven to be involved in EMT (epithelial–mesenchymal transition), a mechanism that sustains invasions and metastasis. Therefore, the inhibition of RAS/MAPK signaling using PD325901, an MEK inhibitor, was correlated with a reduced metastatic progression as an effect of transplantation with stem/progenitor cells [40].

RAF inhibitors were demonstrated to have the capacity to induce homodimerization of ARAF and heterodimerization of BRAF with CRAF and the scaffolding protein *KSR1* in lung cancer cells. In a study, ARAF was determined to be required for MAPK activation in a cell-type-dependent manner in the case of A549 lung cancer cells and ARAF-knockdown cells [41]. This study revealed a novel function for ARAF, which, once in dimer form, activates the MAPK cascade with an impact on sustaining lung cancer cell invasion [41].

Another factor important for signal transduction is represented by the reciprocal inhibition between MAPK and PI3K/AKT. Activated AKT can phosphorylate RAF, determining its inactivation and, by default, inhibiting MAPK signaling. AZD6244-mediated MEK inhibition was correlated with activation of PI3K/AKT as an effect of hyperactivation of ERBB3, subsequently leading to loss of the inhibitory threonine phosphorylation in the juxtamembrane domains of *EGFR* and *HER2* [42]. EGFR signaling pathways are different in normal breast cancer cells (184A1L5) and triple-negative breast cancer cells (MDA-MB-231). Specifically, in the case of 184A1L5 cells, it was emphasized that extracellular regulated MAP kinase (ERK), c-Jun N-terminal kinases (cJNK), and Signal transducer and activator of transcription 3 (STAT3) are triangulated and strongly coupled, while, in MDA-MB-231 cells, STAT3 is only feebly connected to the ERK/p38/JNK pathway [43]. The connection HER–TKIs was proven to have HER2 expression-dependent anti-tumoral effects in breast cancer models, involving JNK and STAT5A/B signaling [44]. A recent study demonstrated the crosstalk between JNK and STAT3 in oral cancer. The mechanism relies on the overexpression of JNK followed by STAT3 phosphorylation at Ser727, and downregulation of STAT3 phosphorylation at Tyr705, with final inhibition of STAT3 activity [45].

Furthermore, mTOR is also involved in complex interactions with MAPK, and other components of JAK/STAT and Notch-1 pathways in solid tumors [46]. Cross-regulation of the MAPK and PI3K is affected by the presence of mutation of *JAK2* (Janus kinase 2, JAK2-V617F) occurring in myeloproliferative neoplasms [47]. Co-inhibition of the PI3K/AKT and RAF/MEK/ERK pathways appears to be constrained for the inhibition of downstream mTOR effector pathways in KRAS mutant cancer [48]. The mTOR complexes are key modulators of cell growth and proliferation, able to respond to GF stimulation (in a similar manner to MAPK) and finally orchestrate protein translation [49]. mTORC1 (mTOR complex 1) is the main representative of the two complexes (the other one being mTORC2) [49,50]. mTORC1 is composed of the mTOR kinase, its associated adaptor protein Raptor (regulatory associated protein of mTOR), mammalian lethal with *SEC13* protein 8 (mLST8), and a proline-rich AKT substrate of 40 kDa (PRAS40) [50,51]. Rheb also proved to downregulate RAF and downstream MAPK signaling [52]. Dual targeted inhibitors of PI3K/mTOR (PF-04691502) in combination with an MEK inhibitor (PD-0325901) were proven to work effectively in ovarian cancer preclinical studies [53].

Alisertib (MLN8237) is a relevant example that brings further evidence of the crosstalk between the signaling pathways. The aurora kinase A inhibitor was demonstrated to have the capacity to induce cell-cycle G2/M arrest, apoptosis, and autophagy via p38 and AKT/mTOR in breast cancer models [54]. Alisertib has important anti-tumoral effects; however, in clinical studies, some side effects were reported, which were manageable for most of the cases [54]. In lung cancer, MEK inhibition was correlated with the activation of EGFR and stimulation of its specific RTK (receptor tyrosine kinases) receptor that has the further capacity to induce a transient inhibition of ERK phosphorylation in BRAF non-V600E, but not BRAF V600E, mutant cells [55]. Contrary, in breast cancer models, a novel tested MEK inhibitor (PD98059) was proven to promote cell migration by increasing the expression level of nuclear β-catenin [56].

For example, the presence or absence of a specific mutation in TP53 was connected with MKK3/MKK6 and with the downstream activated kinase p38 in cancer. TP53 activation via the p38/TGFβ pathway underlines additional implications of different MAPKs in cancer [57]. p38MAPK a is a unique kinase with four different isoforms (α, β, γ, δ), which are expressed differentially in tissues, with α and β isoforms being the most commonly expressed. Thus, p38MAPK activation relies on an MKK3/MKK6 event, which allows the subsequent activation p53. If p53 is mutated in the cell, there is an increased *MKK3* gene expression via NF-Y and NF-κB transcription factors, which subsequently contributes to sustaining a positive feedback loop for the p38MAPK signaling; this can cause stimulatory effects on survival or chemoresistance [58]. This emphasizes that TP53 and p38/MAPK interactions occur at multiple levels. In the case of TP53 wild type, the modulation of growth arrest or apoptosis is done via *p21* gene and *Wip1* [58]. The other player, MKK6, has a dual role, either pro-apoptotic or pro-survival, depending on the cellular and mutational status. Additionally, p38MAPK is also involved in pro-inflammatory signaling by activating STAT3 in the IL-6 (interleukine 6) signaling pathway [58]. Most recently, p38α was demonstrated to induce autophagy, indirectly promoting senescence and protecting cancer cells from chemotherapy-induced apoptosis [59].

As mentioned above, cancer-associated alterations of MAPK signaling do cause a specific resistance to therapy. For example, the resistance to endocrine therapies is primarily facilitated by the molecular modification from estrogen-dependent to estrogen-independent status in ER^+^ (estrogen-receptor positive) breast cancer cells; however, the mechanism via which the MAPK/ERK pathway is involved in estrogen-independent breast cancer is not understood. Peng et al. (2017) were able to demonstrate that *Linc-RoR* (long intergenic non-protein-coding RNA, regulator of reprogramming), a gene that produces a long non-coding RNA regulating the reprogramming of pluripotent stem cells, functions as an onco-lncRNA to specifically promote this estrogen-independent growth of ER^+^ breast cancer. The CRISPR Cas9 (clustered regularly interspaced short palindromic repeats associated protein 9 nuclease) system was implemented for the knockout (KO) of *Linc-RoR* in the MCF-7 cell line. The authors found the following: firstly, *Linc-RoR* initiated an upregulation of phosphorylation in the MAPK/ERK pathway and this in turn activated ER signaling; secondly, the *Linc-RoR* knockout in MCF-7 cells cancelled the induced ERK activation and ER phosphorylation from the estrogen deprivation, and rescue experiments that re-established *Linc-RoR* expression also restored the aforementioned phenotypes. Thirdly, *Linc-RoR* KO-induced repression of MAPK/ERK signaling was at least in part regulated by the ERK-specific phosphatase DUSP7 (dual specificity phosphatase 7). More specifically, the authors claimed that the repression of ERK phosphorylation was caused by the increased protein stability of DUSP7 in the *Linc-RoR* knockout MCF-7 cells [60].

An example of tumor progression through alterations of MAPK signaling was reported in a recent study. Han et al. (2019) used the CRISPR/Cas9 system for fibroblast growth factor-5 (FGF5) knockout in osteosarcoma cells (MG63 and U20S) to determine that FGF5 promotes cell proliferation by activating the MAPK signaling pathway. Since this pathway regulates the proliferative signals from cell surface to nucleus through phosphorylation, the quantification of phosphorylated MAPK proteins was used to demonstrate that FGF5 promotes cell proliferation. Western blot determined that, compared to the controls where there was no recombinant FGF5 added, the expressions of phosphorylated MAPK-associated proteins (p-MEK2, p-ERK, p-Elk-1, and p-MNK1/2) in the FGF5 knockout group were significantly lower. Moreover, these MAPK-associated proteins had their expression levels increased compared to the controls only when the recombinant FGF5 was added. Additionally, FGF5 knockout in the MG63 and U20S cell lines inhibited proliferation, and only the addition of exogenous rFGF5 restored proliferative capacity [61].

## 4. Implications of the Tumor Microenvironment in Regulating MAPK Signaling Pathway

The tumor microenvironment (TME) consists of the peritumoral region characterized by the presence of the tumor, normal and immune cells (e.g., tumor-associated macrophages and tumor-infiltrating lymphocytes), and stroma. Functionally, the TME is an extensive communication network that sustains tumor development through the localized action of different chemokine and cytokines in association with altered signaling pathways [68]. The stroma is a dynamic supportive structure of the TME, consisting mainly of the extracellular matrix, other cells such as fibroblasts and localized immune cells, and histological structures such as blood vessels and connective tissue [69]. The complex structural and immune cellular association in the TME is what confers the tumor with reactivity versus the environment in the form of inflammation and angiogenesis, as the TME allows the localization and signal amplification of all the secreted growth molecules [69].

As mentioned, the TME encompasses many factors important for tumor development. More so, studies point toward the idea that it represents an essential source of resistance to MAPK inhibitors [70]. Associated normal cells of the TME provide the tumor cells with necessary GFs and signal mediators like cytokines [69], while the extracellular matrix (ECM) acts more than a scaffold, as it dictates complex biochemical interactions inside the defined microenvironment. ECM components constantly interact with integrins receptors from the cell surface in the form of two-directional signaling regulated by GFs. These factors modulate the affinity of these interactions, in a process named “inside-out signaling” [71]. The integrin–ECM interactions also modulate the GFs regulated signaling through “outside-in signaling”. The coalescent response of integrin–ECM–GF interactions inside the tumor microenvironment promotes the activation and localization of RAS to the inner membrane. Moreover, ECM regulates intercellular communication, cell junction plasticity, and cell adhesion molecules that interact with a wide range of cytokines/chemokine or growth factors [5,69,72,73]. Among the most studied integrin-mediated signaling effectors in cancer cells are the non-receptor tyrosine kinases focal adhesion kinase (FAK) and Src integrins that are linked to downstream signaling effectors such as the Rac1 GTPase and MAPK [74].

The disassembly of cell-to-cell focal adhesion points is a migratory promoting event commonly observed in cancers, especially during EMT. These focal adhesion points are crucial in mentioning the structural integrity of the tissue, which does not permit cells to detach and migrate in normal physiological conditions. On the other hand, in cancer, RAS was proven to have the capacity to induce disruption of adherent junctions but not tight junctions (Zonula occludens-1 or ZO-1 and occludin) or desmosomal component desmoplakin, while being interconnected with ERK1/2 and PI3K [75]. The urokinase-type plasminogen activator receptor (uPAR) is a glycophosphatidylinositol-anchored cell membrane receptor, related to urokinase (uPA) proteolytic activity retrieved on the cell surface [76]. uPAR is generally connected with tumor dormancy, EGFR is activated by uPAR (ligand-independent mode), sustaining cell proliferation via a mechanism where fibronectin/αvβI integrin and ERK are stimulated [68,76], finally leading to an increased metastatic rate in *RAS*-mutated tumors [76]. A study regarding treatment resistance in cancer who utilized breast cancer two- and three-dimensional (2D and 3D) cell culture models revealed a different response rate; the *HER2*-amplified AU565 cell line was more sensitive to trastuzumab when cultured on top of a 3D lrECM compared to the 2D cell culture condition [77]. The 3D cultures affected ER2 downstream signaling and activated a switch between PI3K–AKT–RAS–MAPK signaling in breast cancer cells lacking *HER2* amplification and overexpression [77].

The TME proved to be an important root of resistance to MAPK pathway inhibitors through macrophage-derived mediator TNFα (Tumor necrosis factor alpha) [70]. Studies proved that the number of tumor-associated macrophages increases in BRAF- and MEK-depleted melanoma cells and BRAF (V600E) melanoma allografts. For head and neck cancer, p38 proved not only to stimulate cell growth but also to promote tumor-induced angiogenesis and lymphangiogenesis [78]. RAS and NF-κB were proven to be involved in ROS (reactive oxygen species) production and inflammation of the tumor stroma as a result of metabolic interaction. The activation of glycolysis and upregulation of protein markers (Caveolin-1, MCT1-solute carrier family 16 member 1, and MCT4) were noted in the case of co-culture of HaCaT and HaCaT *RAS* mutant cells with normal fibroblasts. Cell signaling involving these oncogenes can promote the formation of a stromal–epithelial “lactate shuttle”, which fuels the anabolic growth of cancer cells [79].

Pathological processes, like cancer-related hypoxia, permit cell survival via activation of hypoxia transcriptional programs, comprising HIF-1α (hypoxia-inducible factor 1α), NFĸB, PI3K, and MAPK pathways [80]. Inhibition of MAPK interferes with the transactivation activity of p300/CREB(cAMP responsive element binding protein 1)-binding protein (CBP) by HIF-1*α* and HIF-2*α* that undergo oxygen-dependent degradation [81]. MAPK signaling promotes *HIF-1*α activation, an important bridge related to oncogenesis and activation of neoangiogenesis. This connection is therapeutically valuable and should be considered in developing novel therapies with an effect upon activated *HIF-1*α [81]; moreover, hypoxia regulates not only cancer progression through angiogenesis and metastasis but also the resistance to therapy [80].

Tumor-infiltrating immune cells are important modulators in the tumor microenvironment, involved in a multitude of tumor-promoting functions (EMT, angiogenesis, and immune suppression). EMT is a process that furnishes invasive, migratory, and stem-cell properties for tumor cells, a fact that enables them to disseminate and propagate at distant sites [82]. TGFβ was proven to rapidly activate ERK via RAS, metaphorically presented as “partners in crime”, as they were demonstrated to be critical for specific activation of genes regulating of EMT and cellular motility. These interactions are the main causes in the development of the late disease stages [18]. It is important to mention that the mechanism is reversible, following the removal of TGFβ1, when a constitutively activated RAS signaling pathway is absent in human tumors [83]. As a specific example, in breast cancer, increased expression of RAS has a direct impact upon the downstream mediators (PI3K and/or ERK1/2), an event that is correlated with an unfavorable prognostic [83]. The responsiveness of RAS in the TME frequently implies a phenotypic modification of the tumor cell toward a migratory status, through the loss of cell-to-cell adhesion via EMT and its related effectors in tumor cells [83].

TGFβ1 and the granulocyte macrophage colony-stimulating factor (GM-CSF) are regulated via the MAPK/PI3K axis, while IL-10 (interleukin-10) and CXCL8 (C-X-C motif chemokine ligand 8) are activated via the MAPK/NF-κB axis [84]. Another important example event is represented by the release of IL-6 as an effect of MAPK/NF-κB activation, and its responsive transcription factors STAT3 in *KRAS*-mutant lung tumors. Thus, by blocking IL-6, the lung tumor microenvironment can be reprogrammed to limit tumor development and progression in KRAS tumors [85]. Furthermore, RAS then can indirectly inhibit TGFβ1 through the action of the activated ERK1/2 pathways and promote the stabilization of the EMT process [86]. ERK1/2 could accelerate EMT through its nuclear translocation, where it can regulate the expression of EMT-involved genes [87].

It was demonstrated that the extracellular stresses produce cytokines and chemokines, and that they are related to MAPK activation in pathological conditions. Recent data showed that MAPK activation is crucial in regulating inflammation-associated cancer development. The immune microenvironment represents a source of resistance to MAPK pathway inhibitors also via an increased number of tumor-associated macrophages, and *TNFα* and *MITF* (microphthalmia transcription factor) expression, observed in *BRAF*-mutant melanomas [70]. This correlates with a significant upregulation of *TNFα* expression in the tumor microenvironment, conferring antiapoptotic protection and resistance to MAPK pathway inhibitors through the combined action of TNFα and MITK (melanocytic specific transcription factor), in the case of melanoma cells [70]. The inhibition of NF-κB signaling pathway components like IκB is associated with improvements in the efficiency of MAPK inhibitors [70]. This is the case of lovastatin that targets MAPK and NF-κB pathways, via a mechanism involving death receptors in glioblastoma [67].

## 5. MAPK, Crosstalking Pathways, and Drug Resistance

Cancer drug resistance still remains an important barrier in oncology. The mechanism of resistance can arise prior to or as a direct effect of cancer therapy [88]. This issue can be related to alteration in drug transport mechanisms, mutation and amplification of drug targets, and genetic rewiring through bypassing of targeted pathways or via activation of compensatory ones. In order to increase the patient’s survival rate, different combinational approaches of chemotherapeutics were highlighted for more efficient targeted treatment strategies [89].

In terms of targeted therapies, particular attention is paid to the MAPK inhibitors, not only for the modulation of the apoptotic mechanism but also for their capacity to target cell survival under hypoxic conditions; however, limitations were encountered due to activation of compensatory pathways. Therefore, targeted single agents were improved through the combination of other agents, to prevent the activation of feedback mechanisms responsible for drug resistance [88,89]. In general, most of the resistant cells have stem-like or mesenchymal features [90].

**Figure 2:**
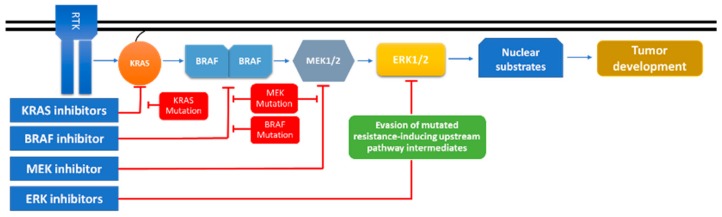
Mitogen-activated protein kinase (MAPK) inhibitor efficiency based on mutational status—cause of resistance and weak spots. Treatment resistance is a reoccurring problem in the case of MAPK pathway inhibitors. The post-treatment acquirement or selection of tumor cells with new mutations renders the treatment useless. In the case of KRAS, BRAF, and MEK inhibitors, mutations in any of these two components can determine therapeutic resistance and relapse. Targeting ERK can become a true Achilles heel in treating cancers with MAPK signaling alterations, as ERK inhibitors target specifically downstream of the signaling cascade, with no regard of the mutational status of the upstream components (e.g., KRAS and BRAF) (KRAS: Kirsten rat sarcoma viral oncogene homolog; BRAF: B-Raf proto-oncogene serine/threonine kinase; ERK:extracellular regulated MAP kinase).

The resistance to cisplatin, irinotecan, and 5-fluorouracil is directly related to MAPK signaling in colorectal cancer, and recent studies demonstrated the p38/MAPK pathway as a relevant effector in affecting the response to therapy and chemoresistance [91]. Even if encouraging outcomes were recorded in early trials with MAPK inhibitors, resistance to therapy inevitably arises. An increased response to therapy can be achieved by combining RAF and MEK inhibitors for the cases that harbor mutant *BRAF* or *KRAS* [92]. These two mutated genes are found in over 30% of all human tumors and in 40% of melanomas [92,93]. Increasing experimental data suggest that the supplementation of a RAF inhibitor along with a small molecule that targets MEK could hold the capacity to delay or overcome cancer drug resistance [92,93]. A schematic overview of the inhibition strategy for the case of MAPK inhibitors is presented in Figure 2.

In BRAF wild-type lung cancer preclinical models, the biological active properties of single or combined therapies for BRAF (dabrafenib), pan-RAF (RAF265), MEK (trametinib), and EGFR/HER2 (lapatinib) were evaluated. It was demonstrated that the combination of trametinib and dabrafenib has the capacity to prevent MAPK activation, reported for the case of single BRAF inhibitors [55]. Meanwhile, for the case BRAF non-V600E mutant lung cancer models, a disabled kinase signaling transduction was observed, where EGFR has the capacity to activate the MAPK cascade. In these circumstances, a combination of MEK and EGFR inhibitors was tested, leading to significant anti-tumor activity [55]. At the same time, co-inhibition of another important RTK representant, FGFR (fibroblast growth factor receptors), and MEK was correlated with an enhanced therapeutic response and minimized acquired resistance in lung cancer [94].

Previous studies on the crosstalk between MAPK pathways and other signaling pathways are represented by the atypical members of the PTK (Protein tyrosine kinase), called pseudophosphatases. Despite this lack of catalytic function, pseudophosphatases were incriminated in various diseases. Mutations inside their catalytic units provide a sustained function at this site, meaning continued or prolonged activation and phosphorylation, mechanistically comparable to the well-characterized example of RAS, which once mutated leads to the sustained activation of the RAS/RAF/MEK/ERK signaling cascade in the tumor cell [95]. This is the case of the STYY (phosphor/serine/threonine/tyrosine-binding protein), which compete with MKP-2 (MAPK phosphatase-2) for binding to ERK1/2, having the competitor role. Furthermore, MK-STYX (mitogen-activated protein kinase/phospho-serine/threonine/tyrosine-binding protein) is associated with tumorigenesis, found to be overexpressed in pediatric Ewing sarcomas [95].

An important role in acquired cancer drug resistance is held by the immune microenvironment [70]. Evidence points toward a protective role of macrophages against MEK inhibitor-induced apoptosis in cancer cells, with this phenomenon counteracted through inhibition of macrophage-derived TNFα [70]. In melanoma, macrophage-derived TNFα is a key factor responsible for the resistance to MAPK inhibitors, acting via MITF, having the capacity to regulate survival and anti-apoptotic genes [70,96]. Dual AURKA (Aurora Kinase A) /MAPK targeting was demonstrated to have the capacity to prevent this resistance [96]. Another important oncogene in melanoma is represented by *BCL2A1* [97]. Co-treatment with BRAF and BCL2 (B-cell lymphoma 2) inhibitors (obatoclax) prevents the intrinsic resistance to BRAF inhibitors, in vitro and in vivo [97]. In addition, in the same pathology (*NRAS*-mutant melanoma cells with wild-type or mutant TP53), an MEK inhibitor (pimasertib) combined with a BCL2 inhibitor (ABT-199) or TP53 stimulator (PRIMA-1^Met^, APR-246) acted in a synergistic model toward apoptosis induction [98].

## 6. MAPK Inhibitors, and Preclinical and Clinical Trial Molecules

**Figure 3:**
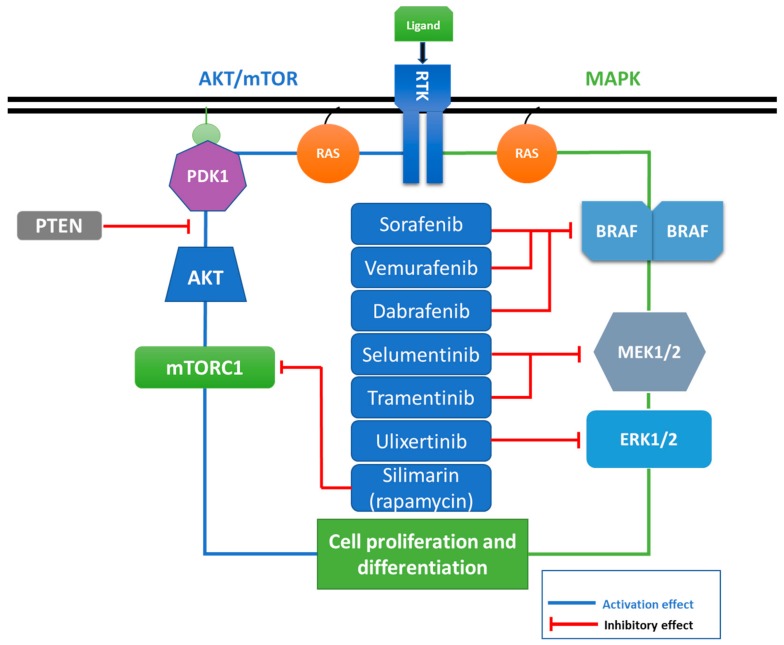
Targeted components of the MAPK and AKT signaling cascades by small-molecule inhibitors in cancer. Effective targeting of the pathway intermediates is an efficient tactic in the case of constitutively activated signaling cascades, such as the MAPK pathway in cancer. Successful inhibition of a step in the cascade impairs the downstream progression of the pathway and its overall aberrant function. Combinations of inhibitors or multi-targeting molecules are being investigated, as they might provide more efficient manipulation of the entire signaling pathway. (MAPK: mitogen-activated protein kinase AKT: v-akt murine thymoma viral oncogene homolog 1).

The description of the mutational landscape in a complex signaling cascade such as MAPK is essential when attempting to outline effective therapies that would target a specific mutated component of the pathway (Figure 3). This is the main direction in which therapies based on inhibitory molecules were developed. Efficient targeting of the aberrantly activated MAPK pathway in cancer is one of the most explored therapeutic approaches. Due to the in-depth characterization of its signaling intermediates, there were major improvements in the treatment of melanoma or lung cancer with the use of generally termed MAPK inhibitors. 

MAPK is considered a key signaling cascade node, which can exhibit, depending on the mutational and cellular context, both tumor-promoting and tumor-suppressor signaling when the case of a mutation arises in one of its effectors. Pro-oncogenic signaling is generally the common case in cancer, often associated with mutations in MAPK pathway effectors (e.g., RAS, BRAF, and MEK1) [28,99]. As mentioned before, some effectors display a dual role, as is the case of p38α, which can act as an anti-proliferative and tumor suppressor in the case of colorectal cancer, while, in some cases, p38α signaling can be responsible for oncogenic-related mechanisms (invasion, inflammation, and angiogenesis) in other cancers [91].

Combinatorial therapies concentrated on the inhibition of multiple checkpoints within interconnected signaling pathways proved to be more efficient, due to the complex nature of crosstalking and activation of compensatory mechanisms toward the same final effector. Table 2 contains information regarding the combined use of preclinical molecules and some molecules that were already introduced clinically, such as lapatinib and crizotinib. The therapeutic efficiency of different combinations of MAPK kinase inhibitors is currently being addressed as a promising improvement to standard therapy.

A great breakthrough was the approval of vemurafenib in 2002, which was the first BRAF inhibitor developed against *BRAF*-mutated melanoma [105]. However, the use of vemurafenib in melanomas with wild-type *BRAF* can act as a stimulator for the MAPK pathway, promoting cell growth and proliferation [35,93]. NSC95397 was demonstrated to reduce cell proliferation in colorectal cancer cells by targeting MKP-1 activity followed by ERK1/2 phosphorylation [66]. Another issue was that, following BRAF inhibitory treatment, some tumors developed resistance. RAF inhibitors (GW5074, L779450, PLX4720, sorafenib, ZM336372) proved to activate RAF dimers, as mutant BRAF cells are dependent on the RAF/MEK/ERK signaling pathway for their growth [106].

As such, ERK re-activation was described to facilitate acquired drug resistance to BRAF inhibitors. In *BRAF*-mutated melanoma models, combined BRAF and MEK inhibition significantly improved clinical outcomes in patients with metastatic melanoma. This combined system emphasizes the important role of PAKs (p21-activated kinases) as pivotal mediators of drug resistance [103].

Activation of autophagy is another offsetting pathway in response to BRAF inhibitors. Key effectors of autophagy activation were proven to be adenosine triphosphate (ATP) secretion, oncogene-induced senescence (OIS), and activation of C-MER proto-oncogene tyrosine kinase (MERTK) [107].

Statins activate cell death-related mechanisms via PI3K/AKT and MAPK/ERK signaling, acting as an effect of the reversion of the metabolic products of the mevalonate pathway or the cholesterol synthesis pathway in breast cancer cells [63]. Therefore, these signaling pathways have an influential function in breast carcinogenesis, growth, and metastasis. Thus, blocking the mevalonate pathway can be a useful strategy in breast cancer prevention or treatment. This is just one example for the compelling preclinical evidence of statin-based therapies showing anticancer effects. This encourages the inclusion in clinical trials of statins as breast cancer adjuvant therapy [108]. Currently, statins are used in women at high risk of breast cancer development (ClinicalTrials.gov identifier: NCT00334542) [109].

Clinical trials represent a bridging step between preclinical research and the clinical implementation of different therapeutic strategies by validating their efficiency, side effects, and safety doses (Table 3). A new MAPK inhibitor that shows promising results in vitro and in vivo would have to pass through a series of clinical trial phases that usually extend upon years of investigations [100,110]. The recent trend in clinical trials involving MAPK inhibitors is the use of combined therapies that either dually target the pathway or inhibit effectors of the compensatory pathways, mainly AKT/mTOR ones [111]. This comes as a response to the treatment resistance that occurs in the case of single-drug therapeutic approaches [112]. The current state of combined therapy using MAPK inhibitors is rather inconclusive, as most studies do not pass the phase II threshold. This is due to a combination of ineffective treatment response rates and high toxicity. Also, the interaction between the used molecules is not well defined in most cases, leading to the question whether simultaneous or intermittent administration is more efficient and less toxic. While the preclinical studies constantly provide innovative molecules and complex combinations with promising results, clinical testing becomes increasingly difficult [111,112].

The therapeutic strategy using MEK inhibitor trametinib in combination with AKT inhibitor afuresertib was poorly tolerated in the case of continuous daily dosing, while intermittent dosing revealed a better tolerability [113].

Non-small-cell lung carcinoma (NSCLC) with *KRAS* mutation is one of the top studied malignant contexts due to acquired resistance, where drug combinations are constantly tested. The combination of selumetinib and erlotinib over monotherapy in *KRAS* mutant and *KRAS* wild-type advanced NSCLC failed to increased patient survival [114]. Another phase I study evaluated the safety and tolerability of ralimetinib, as monotherapy or in combination with tamoxifen for advanced NSCLC patients. The results showed only an increased in the period of stable disease, and no complete or partial response was shown [115]. Dual MAPK pathway inhibition (dabrafenib and trametinib) was proven to have strong anti-tumor activity and good safety profile in patients with *BRAF*-mutant melanoma brain metastases [116], *BRAF*-mutant NSCLC [117], and *KRAS*-mutant-positive NSCLC [118].

Vemurafenib induces significant clinical responses in more than half of patients with previously treated *BRAF* V600-mutant metastatic melanoma, where the median overall survival was approximately 16 months [119]. Cobimetinib (GDC-0973, XL518) was approved for melanoma in tandem with BRAF inhibitor vemurafenib [120]. Vemurafenib and dabrafenib are efficient only in *BRAF* V600-driven tumors [30]. Sullivan et al. presented the first clinical trial with an ERK1/2 inhibitor (BVD-523) for the treatment of patients with advanced solid tumors, where the therapy was proven to be efficient even in *NRAS-* and *BRAF* V600-mutated tumors [30]. Another promising ERK1/2 inhibitor is ulixertinib (BVD-523, VRT752271); this compound has a significant anti-tumoral effect in several cell lines, including those having mutations in the MAPK signaling pathways [121]. This is of particular interest given the recently published review that discussed ERK1/2 regulatory involvement in terms of cellular stress-induced senescence and how this mechanism has the therapeutic potential for regulating cancer [122]. Moreover, this further develops the role that MAPK activity has in terms of cellular stress, more specifically, the discovery that the p38–MAP kinase-activated protein kinase 2 kinase pathway causes cellular response to replicative stress [59].

## 7. MAPK and Natural Bioactive Compounds in Chemoprevention and Chemotherapy

Phytochemicals or related derivative agents were proven to have an important function as therapeutic agents in cancer [129,130,131,132]. The capacity of these secondary plant metabolites to interfere with the expression of coding and non-coding genes is secondly transposed in the modulation of multiple cellular pathways, including MAPK [133,134]; some examples are included in Table 4. MAPK and NF-κB survival pathways were experimentally inhibited using U0126 and caffeic acid phenethyl ester (CAPE) for the reduction of cell growth in pancreatic cells. CAPE activated the apoptosis mechanism only after the inhibition of autophagy [133,135]; this was in a caspase-dependent mode in MIAPaCa-2 cells and in a caspase-independent mode in PANC-1 cells [136]. CAPE is a complex therapeutic agent which targets not only apoptosis pathways, but also those related to EMT and angiogenesis [137]. Furthermore, this study highlighted the importance of the selection of the relevant cell culture model and the knowledge of the features of the cell lines for obtaining valid data [136].

Apigenin is another compound with valuable chemopreventive activities that inhibits the progression and metastasis of choriocarcinoma cells through regulation of the PI3K/AKT and ERK1/2 MAPK signal transduction mechanism [138]. These effects are potentiated by the presence of LY294002 (PI3K/AKT inhibitor) and U0126 (ERK1/2 inhibitor) [138].

Soy leaf components (coumestrol, isotrifoliol, and phaseol) were proven to have important anti-inflammatory effects exercised mainly via TLR (Toll-like receptors)/NF-κB and TLR/MAPK signaling in LPS (Lipopolysaccharide)-induced RAW264.7 macrophages [139]. Coumestrol, a phytoestrogen, holds antiproliferative effects via modulation of MAPK-related genes and an AKT-related compensatory pathway [140]. Quercetin, a flavonol compound, was proven to prevent choriocarcinoma progression via interaction with PI3K and MAPK signal transduction [141]. Moreover, quercetin potentiated the chemotherapeutic effect of cisplatin and paclitaxel in choriocarcinoma cell lines (JAR and JEG3 cell lines) [141].

Kaempferol, another member from the flavanol category, was associated with inhibition of angiogenesis by impairing of HIF-1α and VEGFR2 through a mechanism involving ERK/p38 and PI3K/AKT/mTOR in endothelial cells [142]. Combined treatment of the endothelial cells with kaempferol, along with an ERK inhibitor (PD98059) or p38 inhibitor (SB203580), was reported to potentiate the therapeutic efficacy of kaempferol [142].

Isoflavones were lately considered attractive anti-tumor agents. The effects of novasoy and genistein were evaluated in endometrial cancer cells, where they showed an antiproliferative action, connected with the activation of AKT/mTOR and MAPK signaling pathways [143]. In the same study, it was proven that genistein has the capacity to decrease the expression level for ERα and to increase PR (progesterone receptor) expression level [143]. In melanoma cells, genistein can inhibit cell proliferation, invasion, and migration capacity via FAK/paxillin and MAPK pathways [144]. Additionally, 5,6,7,3’,4’,5’-hexamethoxyflavone (a polymethoxyflavone) was proven to inhibit triple-negative breast cancer cell growth (by targeting MAPK/AKT) and cell-cycle arrest [145].

Resveratrol is a highly disputed natural compound with anti-tumor activity, considered as a potential therapeutic candidate [146]. Resveratrol was investigated for its capacity to specifically activate apoptosis and autophagy in T-cell acute lymphoblastic leukemia (T-ALL) cells, by inhibition of AKT/mTOR/p70S6K/4E-BP1 and activation of p38-MAPK [147].

Escin, a natural mixture of triterpene saponins isolated from *Aesculus hippocastanum*, was proven to have anti-tumor potential, by modulation of apoptosis and autophagy through ROS/p38 MAPK [148].

Next, 21α-methylmelianodiol (21α-MMD), a bioactive derivative from *Poncirus trifoliat*a, has important anti-tumor effects in lung cancer via interference with PI3K/AKT/AMPK and MAPK signaling; it was also associated with MDR (multi-drug resistance) reversal by reduction of P-gp/MDR1 (P-glycoprotein/multidrug resistance protein 1) expressions and its related role, with a further impact on paclitaxel sensitivity [149]. Toosendanin is a natural insecticide that was proven to reverse expression of the EMT markers, via the ERK/Snail pathway in lung cancer models [150].

## 8. Conclusions

The MAPK pathway is the foremost orchestrator of a cancer cell’s response to a wide range of external and internal stimuli. To better understand this orchestrating role that the MAPK pathway has in cancer, the CRISPR Cas9 system can be used to emphasize the functional importance of each pathway member. CRISPR Cas9 knockout will allow for the comparison of the altered gene expression profiles of the interacting MAPK pathway members, when each member is present or absent. This further opens the door to multiple constituents of this complex pathway to be identified as potential targets for drug development. However, the progress was restricted due to the activation of the compensatory pathways in cancer cells. Novel insight upon signal transduction cascades revealed the large degree of crosstalk and redundancy that exists between the different signaling pathways, in tandem with the knowledge of specific mutation status. These mutated genes promote abnormal signaling, affecting the sensibility to a particular treatment, whether mono or multi-therapy inhibitors. Furthermore, we should not underestimate the role of natural compounds and their related derivatives as MAPK modulators and as a starting point for developing new targeted therapies.

Therefore, the MAPK pathway might provide important therapeutic targets due to its capacity to interfere with complex molecular pathways, events demonstrated in many ongoing preclinical and clinical studies that emphasize the next level of combined treatment in the era of precision medicine. The multi-targeted approach is used to prevent the event of resistance upon activation of compensatory pathways related to MAPK. This approach may be the mainstay of new combinations of treatments, with additional agents targeting components of other pathways (e.g., PI3K, AKT, mTOR, or TGFβ), including immune system effectors of the tumor microenvironment.

However, an important aspect in assessing the efficacy of drug combination is the comprehension of how bypassing a pathway step in the presence of a particular mutation can be a key factor in preventing drug resistance. The wide use of genomic and transcriptomic approaches will lead to explaining the functional role of coding and non-coding transcripts, emphasizing their role in different tumorigenesis stages and, therefore, their aspiring nature as therapeutic targets.

## Figures and Tables

**Table 1:** Examples of small molecules tested as mitogen-activated protein kinase (MAPK) inhibitors on in vitro and in vivo studies. EGFR—epidermal growth factor receptor.

Disease	Cell line	Agent	Biological Relevance	Reference
Oral cancer	SCC9 and SCC25	SP600125	Affects cell viability and cell cycle progression via JNK/STAT3	[45]
Melanoma	BRAF mutant cells	PLX4032	Inhibits ERK signaling cascade in a mutant BRAF-selective mode	[35]
	USAC, YUSOC, YUMAC, YUFIC, YUROB, YUGEN, YULAC, MEL501, MEL624, and MEL928 cell lines	GW5074	Inhibition of cRAF without affecting BCL2 and pBad	[62]
Lung and breast cancer cell lines	EGFR and KRAS-mutant cell lines	AZD6244	Activation of PI3K/AKT, negative feedback on ERBB receptors, target ERK	[42]
Breast cancer	MCF-7 and MDA-MB-231 cell lines	PD98059	Promotes invasion, ineffective in breast cancer models, targeting MEK	[56]
	MCF-7, T47D, MDA-MB-231, and BT-549 cell lines	Simvastatin	Anti-tumoral effects by reversing metabolic products of the mevalonate pathway; inhibited MAPK by dephosphorylating sequential cascades of cRAF–MEK1/2–ERK1/2	[63]
	MCF-7 and MDA-MB-231 cell lines	Alisertib	Promotes apoptosis and autophagy by targeting Aurora A via p38 p38/AKT/mTOR pathways	[64]
	MDA-MB-468, BT549, and MDA-MB-231 cell lines	Teriflunomide	Reduce cell proliferation, activation of apoptosis and inhibition of EMT via MAPK	[65]
Colon cancer	SW480, SW620, and DLD-1 cell lines	NSC95397	Reduces cell proliferation via Cdc25 and MKP-1	[66]
Prostate cancer	Mutant mice with prostate specific deletion of *Pten*	PD325901	Activation of RAS/MEK related to PTEN/PI3K/AKT, that conduct the activation of EMT and metastasis	[40]
Myeloid leukemia	TF-1 cells	PD98059	Dual effects on MAPK and AKT pathways in hematopoietic cells	[38]
Glioblastoma	A172, M059J, M059K, and U87, and HEK293T cell lines and nude mice	Lovastatin	Inhibition of NF-κB and ERK but activates JNK; sensitizes TRAIL-induced apoptosis by upregulation of DR5 level via NF-κB inactivation	[67]

JNKs: c-Jun N-terminal kinases; STAT3:signal transducer and activator of transcription 3; ERK: extracellular regulated MAP kinase; BCL2: B cell leukemia/lymphoma 2; pBAD: phosphorylated form of BCL2 associated agonist of cell death; P13k: phosphoinositide-3-kinase; AKT: v-akt murine thymoma viral oncogene homolog 1; MEK: MAP kinse-ERK kinase;mTOR: mechanistic target of rapamycin kinase; EMT: epithelial–mesenchymal transition; ERBB: epidermal growth factor receptor; Cdc25:cell division cycle 25C; MKP-1: dual specificity phosphatase 1; PTEN: phosphatase and tensin homolog; NF-κB: nuclear factor kappa B; TRAIL: TNF superfamily member 10; DR5: TNF receptor superfamily member 10b.

**Table 2:** In vitro and in vivo testing of the effects of MAPK inhibitors.

In Vitro and In Vivo Studies	Compounds	Biological Relevance	Reference
NCI-H1395, NCI-H1755, NCI-H1666, NCI-H508, and SKMEL-28 MRC-5 and 8505C HT-29 and CAL-12T HCC364 and xenograft mouse	Dabrafenib (BRAF inhibitor), vemurafenib (BRAF inhibitor), trametinib (MEK inhibitor), and selumetinib (MEK inhibitor)	Targets critical survival signals in lung cancer, BRAF non-V600E mutant cases	[55]
HCC827, HK2–6, HKE-3, and derivative NCI-H1299 cell lines and xenograft mouse	Dabrafenib, RAF265 (RAF/VEGF inhibitor), trametinib, and lapatinib (EGFR/HER2 inhibitor)	Prevents paradoxical MAPK activation and afford synergistic growth inhibition or additional EGFR blockade in lung adenocarcinoma	[100]
NCI-H2077, RT112, DMS114, and NCI-H520 cells and nude mice	Crizotinib (EML4-ALK) and Trametinib	Prevents drug resistance in in *ALK-*positive tumors	[94]
HCC827, HCC4006, and PC-9, gefitinib-resistant cells, and afatinib-resistant cells	Trametinib and taselisib (*PIK3CA* inhibitor)	Inhibition of MEK and PI3K signaling pathways prevent acquired resistance to EGFR TKIs	[101]
Cell lines sensitive and resistant to therapy and xenograft mouse	PF-04691502 (PI3K/mTOR inhibitor) and PF502 (PI3K/mTOR inhibitor)	RAS signaling as a key mediator of PF502 resistance	[53]
MDA-MB-231	Enterolactone (phytoestrogen)	EMT regulation (inhibiting TGFβ-induced EMT by blocking ERK/NF-κB/Snail)	[102]
Metastatic melanoma cell lines and mice models	PLX4720 (BRAF V600E inhibitors) and PD0325901 (MEK inhibitor)	Drug resistance, via MEK and BRAF, PI3K signaling	[103]
A375, WM266-4, SKMel28, and SKMel2 cells	PD184352 (MEK inhibitor), selumetinib BMS-345541 (NF-κB inhibitor), and SC-514 (NF-κB inhibitor)	Inhibition of TNFα signaling using IκB inhibitors elevated the efficacy of MAPK pathway inhibitors by targeting tumor cell immune microenvironment	[70]
HMEL-B and HMEL-B/M cells	MLN8237 (AURKA inhibitor) and SB415286 (GSK3A inhibitor)	AURKA/BRAF- and AURKA/MEK-mediated resistance mechanism	[96]
Human primary melanocytes, WM1575 and WM3619, and nude mice	PLX4720 and obatoclax (BCL2 inhibitor)	Combined treatment prevents drug resistance and apoptosis	[97]
NRAS-mutant melanoma cells	Pimasertib (MEK inhibitor), ABT-199 (BCL-2 inhibitor), APR-246 (TP53 activator)	Prevent resistance in NRAS-mutant and TP53 mutant by targeting MEK and BCL-2	[98]
PANC-1	Gemcitabine (DNA synthesis inhibitor) and birinapant (IAP antagonist)	Prevent drug resistance activation via FAS and p38	[104]

VEGF: vascular endothelial growth factor; Her2:human epidermal growth factor receptor 2, TKs: Tyrosine kinases; snail: family transcriptional repressor 1; AURKA:Aurora Kinase A; GSK3A: glycogen synthase kinase 3 alpha; BCL2: B cell leukemia/lymphoma 2; IAP: alkaline phosphatase isozyme conversion protein; Fas: cell surface death receptor; EGFR: epidermal growth factor receptor).

**Table 3:** Some relevant examples of application of MAPK in clinical trials.

Clinical Model	Compound	Target Mechanism	Clinical Trial Phase	Observation	Reference
Myelodysplastic syndrome	ARRY-614	p38/Tie2	Phase I (NCT01496495), 2011–2014, completed	Well tolerated, had sufficient activity, and increased therapeutic efficacy	[123]
Solid tumors/multiple myeloma	Trametinib and afuresertib	pan-AKT kinase inhibitor and of MEK1/2	Phase II (NCT01476137) 2011–2017, completed	Intermittent dose; displayed good tolerability	[113]
Advanced cancer (60 participants, non-randomized)	Ralimetinib (LY2228820 dimesylate)	p38 MAPK	Phase I (NCT01393990) 2011–2014, completed	Acceptable safety, tolerability, and pharmacokinetics	[115]
Advanced solid tumors (125 participants—melanoma and lung cancer)	Ulixertinib (BVD-523)	ERK1/2	Phase I dose escalation (NCT01781429) 2013–2018, completed	Responses occurred in patients with *NRAS*-, *BRAF* V600-, and non-V600 *BRAF*-mutant tumors	[30,124]
*KRAS*-mutated and Wild type lung cancer	Selumetinib +/− erlotinib	MEK1/2 and EGFR inhibitor	Phase II (NCT01229150) 2010–2017, completed	No significant improvement related to overall survival	[114]
*BRAF* V600-mutated NSLC	Dabrafenib + trametinib	BRAF and MEK	Phase II (NCT01336634) 2010–2019, active, not recruiting	Important clinical benefit	[117]
*BRAF^V600^*-mutant melanoma brain metastases	Dabrafenib + trametinib	BRAF and MEK MAPK	Phase II (NCT02039947) 2010–2019, completed	Median duration of response was relatively short	[116]
Advanced melanoma *BRAF* V600	Vemurafenib (PLX4032) versus facarbazine chemotherapy	BRAF and methylation agent	Phase 3 trial (NCT01006980) 2011–2016, completed	High rate of response for patient with activating *BRAF* mutations	[119]
Advanced melanoma *BRAF* V600	Vemurafenib and cobimetinib	BRAF and MEK MAPK	Phase I (NCT01271803) 2011–2016, completed	Metabolic alterations rapid after initiation of therapy	[30,125]
Colorectal cancer, NSCLC	Prexasertib (LY2606368) and ralimetinib	Chk1 and P38 MAPK	Phase I (NCT02860780) 2016–2018, completed	Safety profile, target inhibition, and dose-proportional exposure	[126]
*KRAS*-mutant-positive NSCLC	Trametinib (GSK1120212)	MEK1/2	Phase II (NCT01362296) 2011–2014, completed	Trametinib and docetaxel have similar profession free survival	[118]
Adult primary hepatocellular carcinoma	Erlotinib and bevacizumab	EGFR inhibitor and VEGF-A	Phase II (NCT00365391) 2006–2015, completed	Had minimal activity based on evaluated progression-free survival	[127]
Biliary cancer patients	Binimetinib (MEK162)	MEK1/2 inhibitor.	Phase 1 (NCT00959127) 2009–2013, completed	Safe and tolerable, anti-tumor activity in a dose escalation study	[128]

AKT: v-akt murine thymoma viral oncogene homolog 1; MEK: MAP kinse-ERK kinase; BRAF: B-Raf proto-oncogene serine/threonine kinase; ERK:extracellular regulated MAP kinase; EGFR: epidermal growth factor receptor; VEGF: vascular endothelial growth factor.

**Table 4:** Some relevant examples involving the capacity of natural bioactive agents to regulate MAPK in parallel with another related pathway involved in cancer progression and invasion.

Compounds	Disease	Preclinical Model	Molecular Target	Biological Relevance	Reference
Caffeic acid phenethyl ester (CAPE) + U0126	Pancreatic ductal adenocarcinoma	MIAPaCa-2 and PANC-1	↓MAPK and NF-κB expression level	Reduces cell growth by cell-type-specific activation of apoptosis (MIAPaCa-2 caspase-dependent and PANC-1 caspase-independent mode)	[136]
Apigenin	Choriocarcinoma	JAR and JEG3	↓PI3K/AKT and ERK1/2 expression level	Reduces cell viability and migratory capacity; increases apoptosis	[138]
Coumestrol	Prostate cancer	PC3 and LNCaP	↑phosphorylation of ERK1/2, JNK, P90RSK, and P53; ↓phosphorylation of AKT proteins	Inhibits cell proliferation and migration; activates apoptosis	[140]
Quercetin	Choriocarcinoma	JAR and JEG3 cells	↓phosphorylation of AKT, P70S6K and S6; ↑phosphorylation of ERK1/2, P38, JNK and P90RSK proteins	Inhibition of proliferation, cell-cycle progression and invasion; stimulation of ROS production	[141]
Kaempferol	Endometrial malignant transformation	HUVECs andEBM-2	↓phosphorylation of ERK and p38; ERK, p38, Akt; ↓HIF-1α and VEGFR2 proteins	Inhibits angiogenesis	[142]
Genistein	Melanoma	Murine melanoma cell line B16F10	↓ phosphorylation of FAK, paxillin, tensin-2, vinculin, p38, ERK, and JNK proteins	Inhibits the growth and regulates the migration and invasion	[144]
Novasoy and genistein	Endometrial cancer	ECC-1 and RL-95-2 cells	↑phosphorylation of p42/44 in both cell line; ↓ phosphorylation of S6 only in RL-95-2 cells	Reduces cell proliferation and cell-cycle arrest in G2; induces apoptosis	[143]
Resveratrol	T-cell acute lymphoblastic leukemia	T-ALL cell lines, Molt-4 (glucocorticoid resistant) and Jurkat (glucocorticoid resistant)	↓Akt/mTOR/p70S6K/4E-BP1; ↑p38-MAPK	Induces apoptosis and autophagy	[147]
Escine	Osteosarcoma	MNNG, Saos-2, MG-63, U-2OS	↑ p38 expression level	Induces apoptosis and autophagy	[148]
Triterpenoids (21α-methylmelianodiol)	Lung cancer	A549 cells	↓ ERK, p-ERK, JNK, p-JNK, p38, and no effect on p-p38	Targets drug resistance via P-glycoprotein (P-gp)/MDR1-association	[149]
Toosendanin	Lung cancer	A549 and H1975 cells	↓ phosphorylation of ERK; ↓Snail, TGFβ1 expression level	Inhibits TGFβ1-induced EMT and migration, invasion, and adhesion	[150]

ROS: reactive oxygen species; JNKs: c-Jun N-terminal kinases; ERK: extracellular regulated MAP kinase; p38: p38 kinase; AKT: v-akt murine thymoma viral oncogene homolog 1; T-ALL: T-cell acute lymphoblastic leukemia; TGFβ: transforming growth factor beta; VEGFR: vascular endothelial growth factor; HIF: Hypoxia-inducible factors.
